# Effect of arm-ergometry versus treadmill supervised exercise on health-related quality of life and mental health in patients with peripheral artery disease: secondary outcomes from the ARMEX trial

**DOI:** 10.1186/s41687-025-00847-8

**Published:** 2025-02-07

**Authors:** Sandra Magalhães, Mário Santos, Sofia Viamonte, Fernando Ribeiro, Joana Martins, Cristine Schmidt, Henrique Cyrne-Carvalho

**Affiliations:** 1 Department of Physical and Rehabilitation Medicine, Centro Hospitalar Universitário de Santo António, Porto, 4050-011 Portugal; 2 UMIB - Unit for Multidisciplinary Research in Biomedicine, ICBAS - School of Medicine and Biomedical Sciences, University of Porto, Porto, 4050-313 Portugal; 3 Department of Cardiology, Centro Hospitalar Universitário de Santo António, Porto, 4050-011 Portugal; 4 Pulmonary Vascular Disease Unit, Centro Hospitalar Universitário de Santo António, Porto, 4050-011 Portugal; 5 CAC ICBAS-CHUP – Centro Académico Clínico Instituto de Ciências Biomédicas Abel Salazar – Centro Hospitalar Universitário de Santo António, Porto, 4050-011 Portugal; 6 ITR - Laboratory for Integrative and Translational Research in Population Health, Porto, 4050-600 Portugal; 7 Centro de Reabilitação do Norte, Centro Hospitalar de Vila Nova de Gaia/Espinho, Vila Nova de Gaia, 4405-565 Portugal; 8 Institute of Biomedicine – iBiMED and School of Health Sciences, University of Aveiro, Aveiro, 3810-193 Portugal; 9 Department of Angiology and Vascular Surgery, Centro Hospitalar Universitário de Santo António, Porto, 4050-011 Portugal; 10 Research Centre in Physical Activity, Health and Leisure, CIAFEL, Faculty of Sport, University of Porto, Porto, 4200-450 Portugal

**Keywords:** Cardiovascular rehabilitation, Exercise therapy, Intermittent claudication, Mental health, Peripheral artery disease, Quality of life

## Abstract

**Background:**

Peripheral artery disease (PAD) negatively affects walking performance, health-related quality of life (HRQoL) and mental health. Exercise training is recommended as a first-line treatment for PAD, with potential impact on all these outcomes, but the optimal program design is not completely ascertained. The aim of this study was to compare arm-ergometry (AEx) and treadmill supervised exercise training (TEx) on HRQoL and mental health in patients with PAD.

**Methods:**

This was an ancillary study of the ARMEX trial, a single-center, single-blinded, parallel group, randomized clinical trial, enrolling symptomatic PAD patients referred to a cardiovascular rehabilitation program (CRP). Participants were randomized (1:1) to a 12-week AEx or TEx, along with the core components of a CRP (nutritional and psychological support). Participants completed the short form 36 Health Survey and the Hospital Anxiety and Depression scale before and after the intervention. Differences between groups in the change from baseline to the end of the study were analyzed using ANCOVA, adjusted for baseline values, or the Mann-Whitney U test.

**Results:**

Fifty-six patients (66 ± 8.4 years; 87.5% male) were included: AEx (*n* = 28) and TEx (*n* = 28). Physical functioning, role-physical, bodily-pain, general health, mental health and physical component summary (PCS) significantly improved in AEx group. In the TEx group, physical functioning, role-physical, bodily-pain, vitality, social functioning, role-emotional and PCS significantly improved. Role-physical and role-emotional improved more in TEx, with no between-group differences in the other domains. Changes in PCS were significantly associated with changes in walking distances. Hospital Anxiety and Depression scale scores improved in both groups, without between-group differences. This improvement was associated with self-reported walking distance.

**Conclusion:**

Both exercise protocols improved HRQoL and mental health in patients with symptomatic PAD, highlighting exercise-based programs as important treatment strategies for this population.

**Trial registration number:**

ISRCTN54908548 (retrospectively registered).

**Supplementary Information:**

The online version contains supplementary material available at 10.1186/s41687-025-00847-8.

## Introduction

Peripheral artery disease (PAD) is a prevalent condition associated with adverse clinical outcomes, including decreased physical fitness and impaired functional status [1].

Health-related quality of life (HRQoL) is a multidimensional concept that reflects an individual’s subjective assessment of their physical, mental, emotional, and social well-being and functioning [2]. Previous research has shown that patients with PAD experience diminished HRQoL, similar to that of individuals with other cardiovascular conditions [3]. Poor HRQoL predicts long-term survival in this population, providing prognostic value beyond established biomedical risk factors [4]. A recent statement highlighted the importance of incorporating HRQoL assessment in treatment outcomes for all symptomatic PAD patients, to ascertain whether therapies that improve exercise performance also enhance patient’s perception of improvement in the performance of daily life activities [5].

Due to the chronic debilitating nature of PAD, mental health conditions such as depression and anxiety are also more prevalent [6]. The PORTRAIT registry, a multicenter international study, enrolled 1275 patients with PAD and reported a 14.1% prevalence of depression symptoms and 8.3% prevalence of anxiety [6]. Patients at a higher risk of mental health issues were typically younger, female, financially strained, with less social support and worse perceived health status [6]. The increased mental health burden in PAD has been shown to negatively impact outcomes, including health status trajectories, post-revascularization results, and the risk of amputation, long-term mortality, and cardiovascular events [7]. As a first-line treatment, exercise training requires a lifestyle change, and mental health issues can hinder individuals’ ability to adhere to exercise regimens and effectively manage their chronic condition [8]. It has been established that evaluating mental health in clinical practice is crucial, and addressing these conditions should be an integral component of comprehensive PAD management [7]. Therefore, promoting therapeutic strategies that enhance HRQoL and mental health in patients with PAD becomes essential, and supervised walking exercise has previously been demonstrated to improve HRQoL in this clinical setting [9, 10]. A meta-analysis suggested that exercise training may be effective in alleviating anxiety and depression symptoms in patients with coronary heart disease [11]. However, there is a lack of studies in the literature that have evaluated the impact of exercise training on mental health in patients with PAD. Cardiovascular rehabilitation (CR) is a multidisciplinary intervention with well-recognized core components, and a position paper from the European Society of Cardiology recognizes PAD as a qualifying diagnosis for this type of intervention [12]. However, only a small percentage of patients with PAD are currently referred to CR, typically when they have additional cardiovascular conditions [13]. The evidence for CR programs (CRP) in patients with PAD remains limited due to the lack of randomized controlled trials [14].

Several researchers emphasized the importance of conducting high-quality intervention trials that compare different exercise modalities and identify the most effective elements for improving outcomes in symptomatic PAD patients [10, 15]. The ARMEX trial (Arm-ergometry versus Treadmill Supervised Exercise on Cardiorespiratory Fitness and Walking Distances in patients with Peripheral Artery Disease) was a prospective, single-blind, randomized clinical trial comparing arm-ergometry supervised exercise training with a standard treadmill protocol on peak oxygen consumption (VO_2_ peak) and in walking distances among patients with symptomatic PAD [16]. The primary results of this trial indicated that arm-ergometry was noninferior to treadmill training for VO_2_ peak, but treadmill training led to greater improvements in walking distances [16]. However, there is limited data on the impact of these different exercise modalities on HRQoL and mental health. The pain experienced by patients using the standard treadmill protocol has been identified as a potential barrier to adherence [17]. Additionally, evidence suggests that patients with PAD demonstrate better adherence to exercise programs that do not involve pain [18]. Arm-ergometry appears to be a promising modality for improving both HRQoL and mental health in these patients, as it does not cause pain and has been shown to promote pleasurable feelings in this population [19]. Given this background, we aimed to compare the effects of an arm-ergometry supervised exercise training protocol with a treadmill protocol on HRQoL and mental health in symptomatic PAD patients. This study derived from a pre-specified secondary analysis of the ARMEX trial. Additionally, this explanatory analysis sought to assess the relationships between intervention-induced changes in the physical component score of short form 36 Health Survey (SF-36) and walking performance outcomes, as well as the relationships between intervention-induced changes in Hospital Anxiety and Depression scale (HADS) scores and walking performance outcomes.

## Methods

### Study design

The ARMEX trial was a prospective single-center, single-blind, parallel group, noninferiority randomized clinical trial (1:1), comparing the efficacy of arm-ergometry (AEx) versus treadmill exercise (TEx) training on improving cardiorespiratory fitness and walking distances in PAD patients with intermittent claudication over 12-weeks [16]. The current study encompasses a pre-specified secondary analysis from this randomized clinical trial.

PAD patients with intermittent claudication consecutively referred to our hospital’s Cardiovascular Rehabilitation Unit were recruited. Eligible patients underwent a treadmill cardiopulmonary exercise test (CPET) before a final decision regarding final eligibility and randomization was made. The enrollment occurred between January 2017 and April 2023.

Eligible patients were randomized either to an arm-ergometry supervised exercise training (AEx) or a treadmill walking exercise protocol (TEx) over 12-weeks. The study was conducted according to the recommendations of the Declaration of Helsinki and with approval from the Ethics Committee of Centro Hospitalar Universitário de Santo António and School of Medicine and Biomedical Sciences at the University of Porto [2016-053(047-DEFI/046-CES)]. All participants provided written informed consent prior to their inclusion in the study. The current manuscript was organized following the guidelines outlined in the Consolidated Standards of Reporting Trials (CONSORT) Statement for randomized trials of nonpharmacological treatments [20].

### Participants

Adults aged 18 years and above diagnosed with symptomatic PAD and referred to the Cardiovascular Rehabilitation Unit were recruited. The criteria for inclusion and exclusion were previously described [16].

### Randomization and allocation

As previously reported [16], randomization was performed with a computer-based random number generator using a 1:1 allocation ratio for block sizes of four patients. Randomization and allocation were undertaken by a member of the research team (C.S.) who was not directly involved in the recruitment or patients’ assessment.

### Outcomes

#### Health-related quality of life

To evaluate HRQoL the SF-36 was use, considering the eight domain scores. Four of the subscales are related to physical health: physical functioning (PF), role physical (RP), bodily pain (BP) and general health (GH); and the remaining four scales are related to mental health: vitality (VT), role emotional (RE), social functioning (SF) and mental health (MH). Domain scores range between 0 and 100, with higher scores reflecting better QoL. These scores were Z-transformed and weighted to calculate physical component summary (PCS) and mental component summary (MCS) scores [21].

#### Anxiety and depression

Patient-reported symptoms of anxiety and depression were measured using the HADS [22]. This questionnaire comprises seven questions for anxiety and seven questions for depression that score for two different subscales: anxiety (HADS-A) and depression (HADS-D). Each item is answered by the patient on a four-point (0–3) response category so the possible scores ranged from 0 to 21 for each one subscale. Lower scores reflect a lower symptom presence [23].

#### Walking performance outcomes

The assessment of CPET walking outcomes and 6-minute walk test (6MWT) distances was conducted as previously described [16].

### Dropout and adherence rate

Dropout rate was calculated in both groups. Adherence rate was calculated based on the number of sessions performed in relation to the number of sessions planned.

### Interventions

Exercise sessions consisted of twice-weekly sessions comprising 10-min warm-up, 30 to 40 min treadmill intermittent walking or arm-ergometry, resistance exercises and 10-min cooldown, led by a physiotherapist under medical supervision. All patients were monitored with an electrocardiographic telemetry system during the sessions, ensuring that all safety protocols for the intervention were followed. A detailed description of the exercise prescriptions for both intervention groups has been previously reported [16]. Additionally, all patients received nutritional individual counselling and psychological intervention (weekly group sessions) according to the international guidelines for the core components of an outpatient CRP [12]. All patients were encouraged to exercise at home at least twice a week for 30 min. Patients were asked weekly about exercise performed at home. It was made a record of the mode and time of exercise realized. Patients were considered to have performed unsupervised exercise if they completed moderate-intensity exercise for at least 30 min per session for at least 2x/week. Recorded exercise was analyzed as a dichotomous variable (yes or no).

### Statistical analysis

The present study investigated secondary outcomes of the ARMEX trial, thus, no sample size was calculated a priori. The distribution of the data was assessed with the Shapiro-Wilk test and via visual inspection of histograms. Continuous data are reported as mean ± standard deviation, mean differences (95% CI) or as the median (interquartile range) and categorical data as frequencies (percentage). For within-group comparisons, from baseline to 12 weeks, were performed paired-samples t test or Wilcoxon signed-rank test. Differences between groups in the change from baseline to the end of the study were tested using ANCOVA, adjusting for baseline value or Mann-Whitney U test.

To assess the relationships between changes in the physical component score of SF-36, changes in HADS scores, and changes in walking performance outcomes after the intervention, the Pearson correlation test was used for variables with a normal distribution, while the Spearman Correlation test was employed for variables with a non-normal distribution.

Statistical significance of *p* < 0.05 was assumed. IBM SPSS statistics version 29.0 (IBM Corp., Armonk, NY, USA) was used to perform statistical analyses.

The effect size for the outcomes was calculated using appropriate statistical methods. For normally distributed variables, effect size was determined using partial eta squared (η²) from the ANCOVA. For non-normally distributed variables, effect size was calculated based on the Mann-Whitney U test. Additionally, a post-hoc power analysis was conducted using G*Power 3.1 to assess the observed power of the statistical tests performed.

### Data availability

The data associated with the paper are not publicly available but are available from the corresponding author on reasonable request.

## Results

**Fig. 1:**
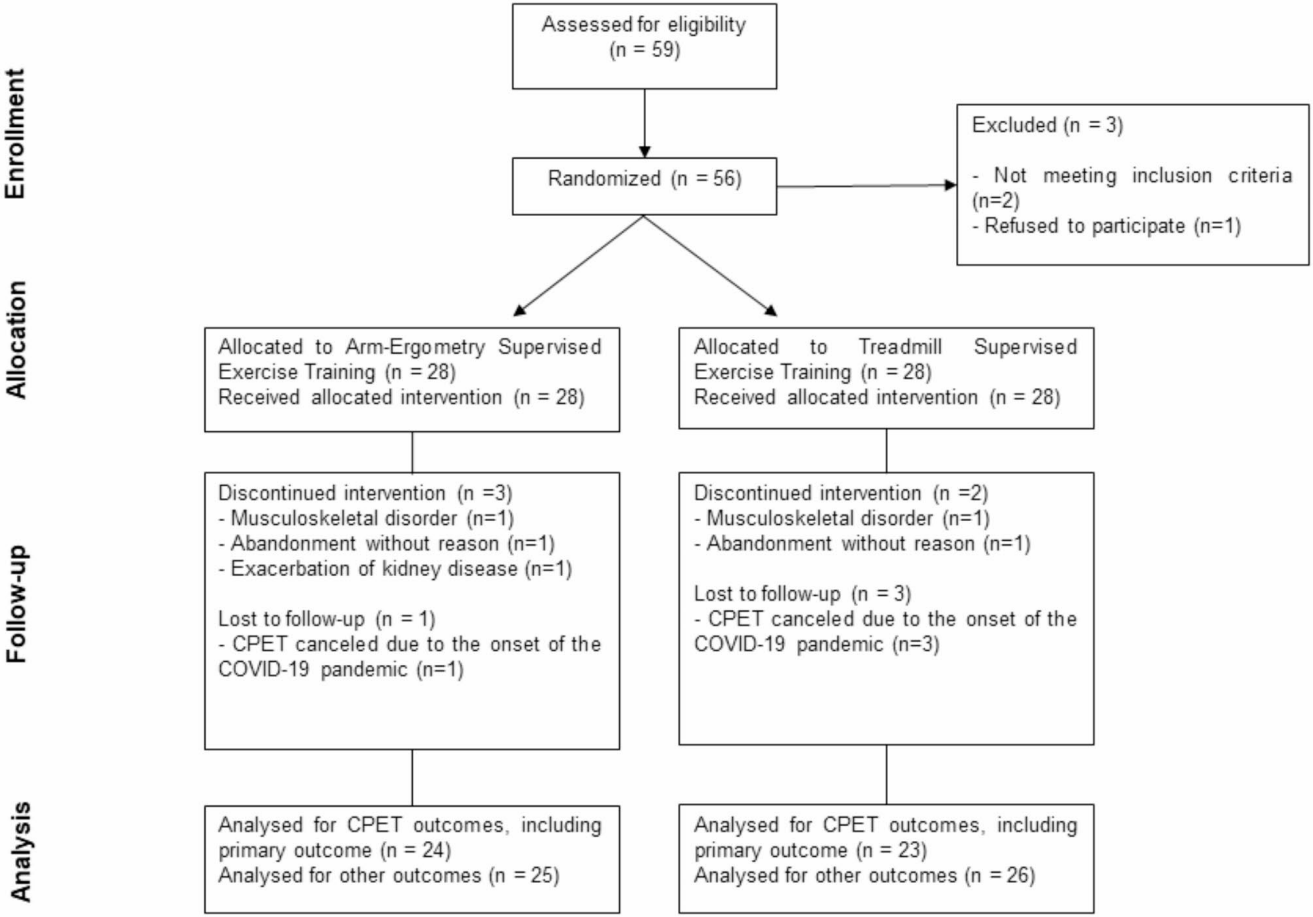
Modified CONSORT flow diagram for individual randomized controlled trials of nonpharmacologic treatments. Abbreviations: CPET = cardiopulmonary exercise test

Fifty-nine patients referred to CR consultation were assessed for eligibility (Fig. 1). One patient refused to participate in the study and two patients did not meet the inclusion criteria. Fifty-six patients (66 ± 8.4 years; 87.5% male) were enrolled and randomized into two groups: AEx (*n* = 28) and TEx (*n* = 28). Of these, 51 patients (AEx, *n* = 25; TEx, *n* = 26) completed the intervention. As shown in Fig. 1, three patients in the AEx group discontinued the exercise program: one due to an exacerbation of lumbar spondyloarthritis, one due to an exacerbation of chronic kidney disease, and one for an unknown reason. In the TEx group, two patients discontinued the intervention: one due to an exacerbation of hip osteoarthritis, and one for an unknown reason.

Exercise session adherence was 91.3% (AEx: 86.9% vs. TEx: 91.7%; *p* = 0.311). The percentage of patients who complied with the recommendation to exercise at home was also similar in both groups (AEx: 76%; TEx: 69.2%; *p* = 0.59). No adverse events were reported during the exercise sessions for any of the patients included in this analysis. Baseline participants’ characteristics and between-group comparisons are described in Table 1.

**Table 1:** Baseline characteristics overall and for the two exercise groups

Variable	Overall *n* = 56 Mean ± SD or *n* (%)	Arm-ergometry *n* = 28 Mean ± SD or *n* (%)	Treadmill *n* = 28 Mean ± SD or *n* (%)
**Age**,** years**	66 ± 8.4	67 ± 7.8	64 ± 8.9
**Male**,** %**	49 (87.5)	25 (89.3)	24 (85.7)
**Lowest resting ABI**	0.66 ± 0.13	0.69 ± 0.11	0.62 ± 0.15
**Prior leg revascularization**,** %**	12 (21.4)	3 (10.7)	9 (32.1)
**Comorbidities**,** %**			
*Coronary Heart disease*	26 (46.4)	15 (53.6)	11 (39.3)
*Cerebrovascular disease*	4 (7.1)	2 (7.1)	2 (7.1)
*Heart Failure*	5 (8.9)	3 (10.7)	2 (7.1)
*COPD*	5 (8.9)	3 (10.7)	2 (7.1)
**Cardiovascular risk factors**,** %**			
*Dyslipidemia*	47 (83.9)	22 (78.6)	25 (89.3)
*Diabetes mellitus*	25 (44.6)	13 (46.4)	12 (42.9)
*Hypertension*	48 (85.7)	22 (78.6)	26 (92.9)
*Physical inactivity*	43 (76.8)	24 (85.7)	19 (67.9)
*Obesity*	10 (17.9)	5 (17.9)	5 (17.9)
*Smoking status*			
Current	17 (30.4)	10 (35.7)	7 (25)
Previous	34 (60.7)	17 (60.7)	17 (60.7)
Never smoked	5 (8.9)	1 (3.6)	4 (14.3)
**Medication**,** %**			
*Acetyl-salicylic acid*	47 (83.9)	22 (78.6)	25 (89.3)
*Statins*	56 (100)	28 (100)	28 (100)
*Beta-blockers*	35 (62.5)	21 (75)	14 (50)
*ACEi /ARB*	43 (76.8)	21 (75)	22 (78.6)
*Antiplatelet agent*	18 (32.1)	10 (35.7)	8 (28.6)
*Anticoagulants*	10 (17.9)	7 (25)	3 (10.7)
*Pentoxifylline*	14 (25)	7 (25)	7 (25)
*Cilostazol*	6 (10.7)	2 (7.1)	4 (14.3)

*Abbreviations* ABI = ankle-brachial index; ACEi = angiotensin receptor blockers; COPD = chronic obstructive pulmonary disease; SD = standard deviation

**Fig. 2:**
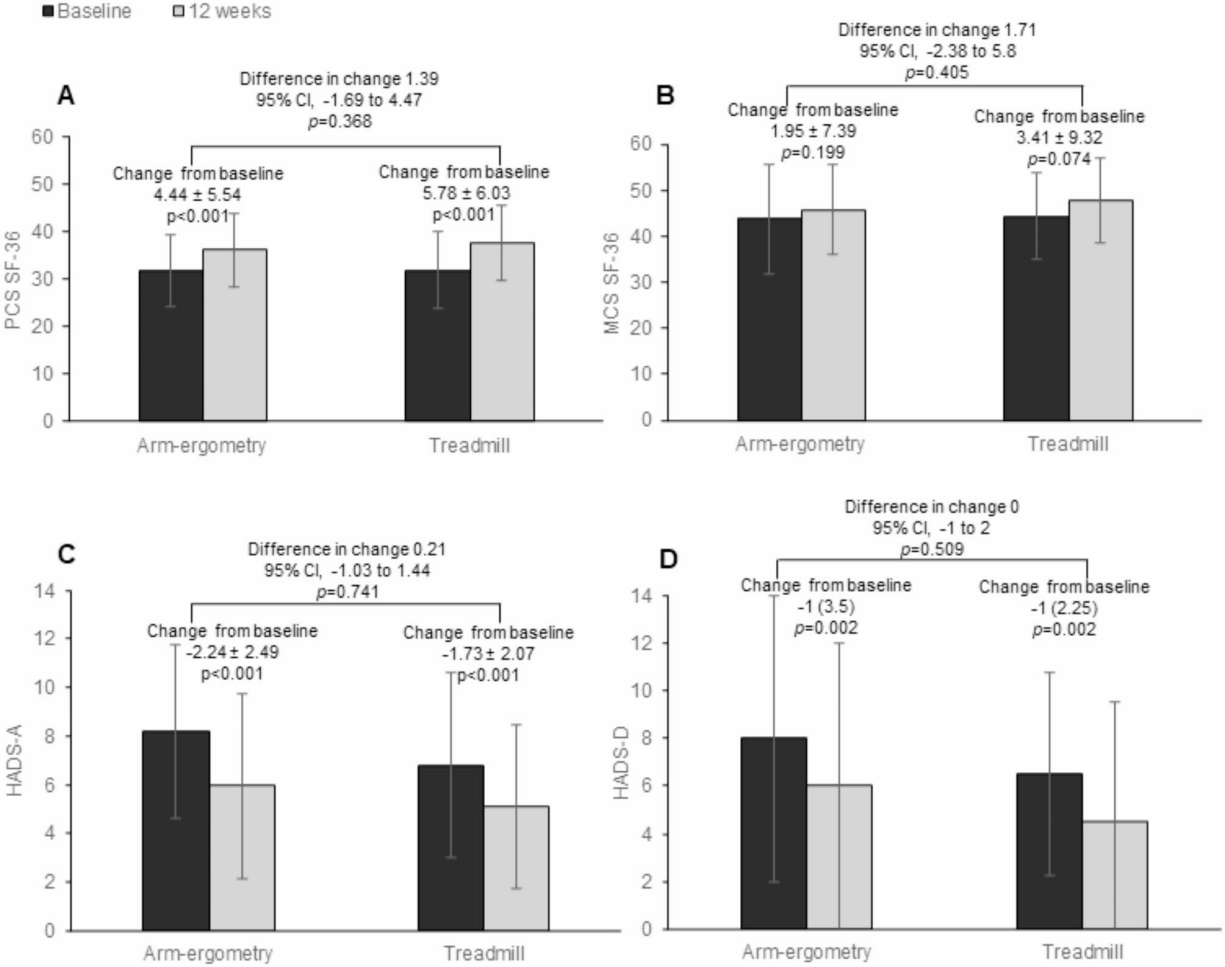
Change in PCS of SF-36 (**A**), MCS of SF-36 (**B**), HADS-A (**C**) and HADS-D (**D**) from baseline to the end of the 12-week exercise program. Data are presented as mean ± SD, mean (95% CI), or median (IQR). Abbreviations: SF-36 = Short form 36 health survey version 2; PCS: physical component summary score; MCS: mental component summary score; HADS = Hospital Anxiety and Depression scale; HADS-A – anxiety score; HADS-D – depression score

At baseline, in both groups, the four domains of the SF-36, related to physical health (PF, RP, GH and BP) had lower scores than the four subscales related to mental health (VT, SF, RE and MH). These results are depicted in Table 2. After the 12-week exercise training intervention, PF, RP, BP, GH and MH increased significantly in the AEx group, and PF, RP, BP, VT, SF and RE increased significantly in the TEx group. RP and RE improved more in TEx, with no between-group differences in the other domains (Table 2; Fig. 2). The PCS increased in both groups, without between-group differences (1.39; 95% CI, -1.69 to 4.47; *p* = 0.368) (Table 2; Fig. 2); the MCS remained unchanged in both groups, without between-groups difference (1.45; 95% CI, -3.29 to 6.20; *p* = 0.541). Change in PCS was significantly associated with changes in CPET pain-free walking distance (PFWD) (*r* = 0.294, *p* = 0.043), 6MWT maximal walking distance (MWD) (*r* = 0.351, *p* = 0.012) and Walking Impairment Questionnaire distance (WIQd) (*r* = 0.312, *p* = 0.026).

**Table 2:** Effects of arm-ergometry and treadmill exercise training on SF-36v2 and HADS and groups differences

	Arm-ergometry *n* = 25			Treadmill *n* = 26			Between-group Difference in Change^a^	*p* value^a^	Effect Size (Partial Eta Squared η²)	Observed Power
	Baseline	Final	Change from baseline	Baseline	Final	Change from baseline				
**SF-36**										
*Physical functioning*	41.82 ± 15.5	53.8 ± 16.41*	11.98 ± 18.75	44.62 ± 18.81	55.58 ± 18.13*	10.96 ± 13.71	0.23 (-8.1–8.48)	0.955	0.00	0.05
*Role-physical*†	0 (50)	25 (100)*	0 (25)	0 (25)	37.5 (81.25)*	25 (50)	25 (0–25)††	0.022^b^	0.32	0.19
*Bodily-pain*†	41(40)	52 (36.5)*	11 (24.5)	41 (30.5)	54.5 (33)*	10 (19.5)	1.75 (-5.5–14)††	0.529^b^	0.09	0.06
*General health*	36.88 ± 14.45	42.72 ± 13.26*	5.84 ± 12.09	44.73 ± 18.7	45.96 ± 20.72	1.23 ± 15.43	2.25 (-5.33–9.83)	0.553	0.01	0.05
*Vitality*†	45 (17.5)	50 (17.5)	5 (12.5)	45 (20)	55 (22.5)*	5 (15)	5 (-5–10)††	0.281^b^	0.15	0.08
*Social functioning*†	62.5 (31.25)	75 (43.75)	12.5 (18.75)	62.5 (25)	75 (27.67)*	12.5 (25)	0 (-12.5–12.5)††	0.779^b^	0.04	0.05
*Role-emotional*†	33.33 (100)	33.33 (100)	0 (0)	50 (34)	83.5 (41.7)*	16.5 (33.33)	0 (0–33.3)††	0.045^b^	0.28	0.16
*Mental health*†	52 (30)	68 (24)*	4 (12)	56 (32)	64 (29)	0 (9)	0 (-8–4)††	0.522^b^	0.09	0.06
*Physical component summary*	31.82 ± 7.64	36.25 ± 7.83*	4.44 ± 5.54	32 ± 8.09	37.78 ± 7.99*	5.78 ± 6.03	1.39 (-1.69–4.47)	0.368	0.02	0.05
*Mental component summary*	43.81 ± 11.87	45.76 ± 9.82	1.95 ± 7.39	44.42 ± 9.4	47.83 ± 9.23	3.41 ± 9.32	1.71 (-2.38–5.8)	0.405	0.02	0.05
**HADS**										
*HADS – A*	8.2 ± 3.58	5.96 ± 3.8*	-2.24 ± 2.49	6.81 ± 3.78	5.08 ± 3.37*	-1.73 ± 2.07	0.21 (-1.03–1.44)	0.741	0.01	0.05
*HADS – D*†	8 (6)	6 (6)*	-1 (3.5)	6.5 (4.25)	4.5 (5)*	-1 (2.25)	0 (-1–2)††	0.509^b^	0.09	0.06

†Variables with non-normal distribution; Wilcoxon signed-rank test and Mann-Whitney U test were performed for within-group and between-group comparisons, respectively; data are median (interquartile range). ††Hodges-Lehmann estimate (estimate confident interval across groups)

^a^*p* value from ANCOVA model, adjusting for baseline value, for between-group difference in change.^b^*p* value from Mann-Whitney U test for between-group difference in change

Data are mean ± SD or mean (95% CI), or †median (interquartile range). *Significantly different from baseline, *p* < 0.05

*Abbreviations* SF-36 = Short form 36 health survey version 2; HADS = Hospital Anxiety and Depression scale; HADS-A – anxiety score; HADS-D – depression score

HADS-A and HADS-D scores improved in both groups, without between-group differences (0.21; 95% CI, -1.03 to 1.44; *p* = 0.741 and 0; 95% CI, -1 to 2; *p* = 0.509, respectively) (Table 2; Fig. 2). Changes in HADS-A and HADS-D were associated with changes in WIQd (*r* = -0.352, *p* = 0.011 and *r* = -0.353, *p* = 0.011, respectively).

## Discussion

At baseline, the SF-36 domains related to physical health exhibited lower scores compared to the mental health domains in both intervention groups, consistent with findings from previous studies conducted on PAD population [24]. Few studies have investigated the HRQoL following a 12-week exercise program and reported on the SF-36 domain scores among patients with PAD. We showed a significant improvement in the four physical health domains (PF, RP, BP and GH) as well as in one mental health domain (MH) following a 12-week arm-ergometry exercise program. Two previous trials have evaluated HRQoL after arm-ergometry exercise training in PAD patients. One study [25], conducted over 6 weeks, showed a significant enhancement of PF and RF domains. In the other study [26], at the 6-week follow-up, no improvement was observed in any domain, but after 24 weeks, significant enhancements were found in PF, BP, GH, VT and MH. To the best of our knowledge, this is the first study to report these results after a 12-week arm-ergometry exercise training; early improvements in HRQoL domains are crucial, as they could significantly impact patient exercise adherence.

In TEx group, a significant improvement in three physical health domains (PF, RP, BP) and three mental health domains (VT, SF, RE) was observed. A meta-analysis encompassing randomized controlled trials comparing exercise versus usual care in PAD patients revealed improvements after a 12-week treadmill exercise in PF, RP and VT domains (2 trials) [9]. A significant increase in most SF-36 domains within both intervention groups was observed in the present trial. These positive results may be related to the incorporation of balance and coordination exercises during the warm-up and cool-down phases of the rehabilitation exercise program, as well as resistance training. A previous trial involving PAD patients demonstrated that the four most significant predictors of the physical function subscale of HRQoL were all patient-based measures of physical function: WIQ speed score, history of stumbling while walking, WIQ stair climbing score, and activities of daily living, specifically bathing [24]. Therefore, incorporating balance and coordination exercises along with resistance training may reduce the risk of falling, enhance autonomy, improve walking speed, and enhance stair climbing abilities, all of which can significantly contribute to improving HRQoL in this population.

The PCS showed a statistically and clinically [27] meaningful improvement in both intervention groups, whereas the increase in MCS did not reach statistical significance. Other studies support our findings [10]. To the best of our knowledge, this is also the first study that compared the HRQoL of PAD patients who performed an arm-ergometry exercise training protocol versus a treadmill protocol. Only RF and RE domains improved more in TEx group, but we must highlight that no significant between-group differences were observed in PCS and MCS subscores. Arm-ergometry appears to be an alternative exercise modality that can enhance HRQoL in the PAD population. The change in the PCS after exercise training correlated with treadmill pain-free walking distance but showed stronger associations with the 6-minute maximum walking distance and self-reported walking distance in the WIQ questionnaire. These results are consistent with previous findings, which suggest a stronger correlation between the improvement in HRQoL and the enhancement in walking performance observed in the 6-minute walk test and the self-reported distance in the WIQ questionnaire [28, 29]. The 6-minute walk test is potentially more reflective of typical walking activities performed daily compared to walking on a treadmill [28, 29].

HADS-A and HADS-D scores improved significantly in both groups after the intervention, without differences between groups. The association of mental symptoms of anxiety and depression with cardiovascular disease is widely recognized and affects prognosis and adherence to treatment [30]. Some reports have also shown a high prevalence of emotional distress in PAD patients, possibly influencing prognosis [31, 32]. Few studies have reported the impact of exercise interventions on anxiety and depression in the PAD population. A previous trial demonstrated no improvement in HADS scores after a community CR program [33]. However, unlike our study, that program did not include a specialized psychological evaluation and intervention [33]. One trial pointed out that the minimal clinically important difference for both HADS anxiety and depression scores, in patients with cardiovascular disease is 1.7 [34]. In present trial, a higher increase was found in both intervention groups for anxiety and depression scores. This improvement was related to the enhancement in self-reported walking distance. Therefore, the improvement in mental symptoms appears to be more closely linked to the individual’s perception of enhanced walking ability in daily life rather than to more objective measures of walking performance.

### Limitations

This was a secondary analysis, and as such, our sample size was determined based on the expected improvement in the primary endpoint (VO_2_ peak) of the ARMEX trial and not specifically calculated for these outcomes. The small sample size for evaluating these outcomes and the overrepresentation of males must be addressed, as they limit the generalizability of the results. The male predominance is a significant concern, as it may not fully reflect the outcomes of the broader population, including females with PAD, for whom sex-based differences in response to supervised exercise therapy have been reported in the literature [35]. Therefore, the findings of this study may not be fully applicable to women or other demographic groups, such as older adults or those from different ethnic backgrounds.

For evaluating HRQoL, we used a generic instrument. However, incorporating a disease-specific survey instrument, such as the short version of the Vascular Quality of Life Questionnaire (VascuQoL6), would have provided a more accurate and relevant assessment, in accordance with recent guidelines [14]. We did not assess the improvement in autonomy in activities of daily living or the risk of falling. Future studies should incorporate performance tests and/or patient-reported outcome measures that address these factors, as they are crucial determinants of HRQoL in this population. Other mental health concerns, such as cognitive performance, sleep disturbances, and attention-deficit/hyperactivity disorders, are also recognized as important outcomes but were not addressed in this study. Future research should involve longer follow-up periods and focus on recruiting a more diverse population, including both sexes, a broader age range, and different ethnic and socioeconomic groups, to enhance the generalizability of the findings. Moreover, it is important to consider factors such as patient preference, exercise adherence, and the cost-effectiveness of these interventions, as these elements are essential for maximizing long-term adherence and ensuring the widespread application of exercise programs for this population.

## Conclusion

Both modes of exercise effectively enhanced HRQoL and reduced anxiety and depression levels in PAD patients, with greater improvements in the physical component.

Assessing and enhancing HRQoL along with addressing mental health concerns are crucial for improving patients’ adherence to rehabilitation programs and effectively manage their chronic disease. Hence, both exercise modalities proved to be effective in improving various outcomes, serving as viable treatment options that enhance bodily functions and increase levels of activity and participation in this patient population.

It is important to note that the improvements in HRQoL and mental health outcomes observed in this study are exploratory and may have generalizability limitations. These findings should be interpreted with caution, and future research should investigate these findings further in diverse healthcare settings and populations to determine their broader applicability and ensure relevance across different patient demographics.

## Electronic supplementary material

Below is the link to the electronic supplementary material.

Supplementary Material 1

## Data Availability

The data associated with the paper are not publicly available but are available from the corresponding author on reasonable request.

## Abbreviations

PAD: Peripheral artery disease
HRQoL: Health-related quality of life
AEx: Arm-ergometry exercise training
TEx: Treadmill exercise training
CRP: Cardiovascular rehabilitation program
PCS: Physical component summary
CR: Cardiovascular rehabilitation
VO_2_ peak: Peak oxygen consumption
SF-36: Short form 36 Health Survey
HADS: Hospital Anxiety and Depression scale
CPET: Treadmill cardiopulmonary exercise test
CONSORT: Consolidated Standards of Reporting Trials
PF: Physical functioning
RP: Role physical
BP: Bodily pain
GH: General health
VT: Vitality
RE: Role emotional
SF: Social functioning
MH: Mental health
MCS: Mental component summary
HADS-A: HADS anxiety score
HADS-D: HADS depression score
6MWT: 6-minute walk test
PFWD: Pain-free walking distance
MWD: Maximal walking distance
